# Bridging the data–power paradox: a conceptual–empirical analysis of solar-powered digital health systems in low-resource settings

**DOI:** 10.3389/fdgth.2026.1848840

**Published:** 2026-05-29

**Authors:** Md Shafiqur Rahman Jabin, Ronald Danny Nyatuka

**Affiliations:** 1 Department of Medicine and Optometry, eHealth Institute, Linnaeus University, Kalmar, Sweden; 2 Centre for Digital Innovations in Health and Social Care, Faculty of Health Studies, University of Bradford, Bradford, United Kingdom; 3 School of Computing and Engineering Sciences (SCES), Strathmore University, Nairobi, Kenya

**Keywords:** distributed health systems, health system integration, health systems strengthening, healthcare digitization, implementation science, infrastructure resilience, rural healthcare delivery, service continuity

## Abstract

**Background:**

Digital health technologies are increasingly promoted as key enablers of health system strengthening in low-resource settings. However, their effectiveness is often constrained by inadequate infrastructure, particularly unreliable energy supply. This misalignment between digital innovation and infrastructural readiness can be conceptualized as the “Data–Power Paradox,” whereby investments in digital health systems are undermined by unreliable electricity and connectivity.

**Objective:**

This study aims to examine the role of energy infrastructure as a foundational enabler of digital health systems and to develop a conceptual–empirical model for integrating solar-powered energy solutions into digital health architectures in low-resource settings.

**Methods:**

A conceptual–empirical approach was adopted, combining secondary analysis of data from the Bright Health feasibility study conducted in Kenya and Ethiopia with a targeted narrative review of the literature. Qualitative insights from healthcare facilities and stakeholders were analyzed thematically, while quantitative indicators related to infrastructure and system performance were interpreted descriptively. A systems-thinking framework was applied to examine the interdependencies between energy reliability, digital system functionality, and health service delivery.

**Results:**

Findings indicate that unreliable energy supply significantly disrupts digital health system performance, leading to system downtime, fragmented data, and reliance on manual processes. Facilities supported by more stable energy sources, particularly solar-powered systems, demonstrated improved system uptime, enhanced data continuity, and greater consistency in digital workflows. The analysis further shows that offline-first system design, when combined with hybrid connectivity and reliable energy infrastructure, can mitigate the effects of infrastructural constraints. These insights informed the development of a solar-powered digital health system architecture that integrates energy, connectivity, and data management components.

**Conclusions:**

Energy infrastructure is a critical determinant of the success of digital health systems in low-resource settings. Addressing the Data–Power Paradox requires integrated approaches that align digital health investments with reliable energy solutions. The proposed solar-powered digital health architecture provides a scalable and resilient model for improving healthcare delivery and advancing equitable access to digital health services.

## Introduction

### The data–power paradox in global health

Over the past two decades, digital health technologies have been increasingly promoted as critical enablers of health system strengthening, particularly in low- and middle-income countries (LMICs). Tools such as electronic medical records (EMRs), telemedicine platforms, and mobile health applications have demonstrated potential to improve clinical decision-making, enhance data continuity, and support more efficient service delivery (1, 2). Despite these advancements, the impact of digital health interventions in many resource-constrained settings has remained limited and inconsistent.

A fundamental yet underexplored challenge underlying this gap is the “Data–Power Paradox”. This paradox reflects a structural misalignment in which substantial investments are made in digital health technologies, while the foundational infrastructure required to sustain them, particularly reliable electricity and connectivity, remains inadequate. As a result, digital systems frequently become underutilized or non-functional, especially in rural and peri-urban healthcare facilities where infrastructure deficits are most pronounced (3).

Evidence suggests that a significant proportion of healthcare facilities in sub-Saharan Africa operate without reliable electricity, with some lacking any form of power supply altogether. Even in settings where digital tools have been deployed, frequent power outages and unstable connectivity disrupt system functionality, leading to fragmented patient records, delays in care delivery, and inefficiencies in clinical workflows (4, 5). These challenges highlight a critical disconnect between technological innovation and infrastructural readiness.

Insights from health information technology (HIT) research further underscore the importance of system dependencies. Studies have shown that digital health systems are inherently complex and highly sensitive to disruptions in their operating environment, including hardware failures, software issues, and infrastructure instability (6, 7). Even in high-resource healthcare systems, such disruptions can compromise data integrity and patient safety. In low-resource settings, where infrastructure constraints are more severe, these risks are amplified, rendering digital health systems particularly vulnerable to failure.

The Data–Power Paradox, therefore, represents not merely a technical issue but a systemic challenge that undermines the effectiveness of digital health investments. Addressing this paradox requires a shift from viewing digital health as a standalone intervention to recognizing it as part of an integrated infrastructure ecosystem in which energy plays a foundational role.

### Link between energy and digital health systems

Reliable energy access is a critical yet often overlooked determinant of health system performance and digital health functionality. Energy underpins the operation of essential medical equipment, supports cold chain systems for vaccines, enables lighting for clinical procedures, and sustains digital infrastructure such as servers, computers, and communication networks (8, 9). Without a consistent power supply, the functionality of these systems is compromised, directly affecting the quality and continuity of care.

In the context of digital health, energy reliability becomes even more crucial. Digital systems depend on continuous power to ensure data availability, system uptime, and secure information exchange. Interruptions in power supply can lead to system shutdowns, data loss, and delays in accessing patient information, thereby disrupting clinical workflows and decision-making processes (1). In many rural healthcare settings, these disruptions lead to a return to paper-based systems, further exacerbating inefficiencies and data fragmentation.

Solar energy has emerged as a promising solution to address energy gaps in healthcare facilities, particularly in off-grid and underserved regions. Solar-powered systems offer a decentralized and sustainable source of electricity, enabling health facilities to maintain operations independently of unreliable national grids (8, 10). Evidence from multiple settings indicates that solar electrification can improve service availability, extend operating hours, and enhance the reliability of digital health systems (11–13).

The integration of solar energy with digital health infrastructure represents a critical opportunity to overcome the limitations imposed by the Data–Power Paradox. By ensuring energy reliability, solar-powered systems can support the continuous operation of digital health systems, facilitate real-time data management, and enable resilient healthcare delivery in resource-constrained environments. Despite growing recognition of this potential, there remains limited empirical and conceptual work that systematically examines the interplay between energy systems and digital health infrastructure.

### Study aims

This study seeks to address this gap by exploring the role of solar-powered infrastructure as a foundational enabler of digital health systems. Drawing on empirical insights from the Bright Health feasibility study conducted in Kenya and Ethiopia, the paper develops a conceptual framework to understand how energy reliability influences digital health functionality and health system performance. In doing so, it contributes to a more integrated understanding of digital health implementation, emphasizing the need to align technological innovation with infrastructural readiness in order to achieve sustainable and equitable health outcomes.

## Methods

### Study design

This study employed a conceptual–empirical hybrid design to examine the role of energy infrastructure as a foundational enabler of digital health systems in low-resource settings. The approach combined secondary analysis of empirical data with systems-level conceptual synthesis, allowing the study to bridge real-world observations with theoretical and policy-relevant insights. Such approaches are increasingly adopted in digital health research to address complex, multi-layered system challenges that cannot be fully captured by purely quantitative or qualitative designs (1, 3).

The study builds on data generated from the Bright Health feasibility study conducted between 2025 and 2026 in Kenya and Ethiopia. That study used a mixed-methods design to assess the technical feasibility, economic viability, and health system readiness of a solar-powered, offline-compatible digital health solution. In the present analysis, the empirical findings were not reproduced in full; instead, selected insights were reinterpreted through the conceptual lens of the “Data–Power Paradox,” which refers to the misalignment between digital health investments and the availability of reliable foundational infrastructure such as electricity and connectivity.

The Bright Health feasibility study was conducted across 12 rural and peri-urban healthcare facilities in Kenya, with additional sites included in Ethiopia as part of the broader multi-country study. The facilities were purposively selected to capture variability in infrastructure conditions, particularly regarding energy access, connectivity, and digital health system readiness. Data were collected from key stakeholders, including facility managers, healthcare providers, and technical personnel involved in digital health operations.

### Data sources

The empirical foundation of this study was derived from the Bright Health feasibility study, which was conducted across rural and peri-urban healthcare facilities in Kenya and Ethiopia. Data were collected using a mixed-methods approach that included key informant interviews, focus group discussions, and structured observational assessments. Key informant interviews were conducted with healthcare workers and facility leadership to capture insights on technical feasibility, economic considerations, and system readiness. Focus group discussions were used to explore shared experiences and stakeholder perspectives, while observational surveys assessed facility-level infrastructure, including power supply reliability, connectivity, and availability of digital systems. Participants and facilities were selected using purposive sampling to ensure representation of diverse implementation contexts, including variations in energy access (grid-dependent and off-grid facilities), ICT infrastructure, and levels of digital health adoption.

To support conceptual development and contextualization, a targeted narrative review of the literature was undertaken. The review focused on digital health implementation in low-resource settings, energy access in healthcare systems, and health information technology failures related to infrastructure dependencies. Searches were conducted using databases such as PubMed, Scopus, and Google Scholar, as well as institutional repositories from organizations including the World Health Organization and the World Bank. Relevant peer-reviewed articles, policy reports, and global frameworks were selected for their relevance to the study objectives and contribution to understanding the interaction between infrastructure and digital health systems.

Data inclusion was guided by relevance to the study objectives, with priority given to facilities and participants providing insights into energy reliability, digital system usage, and implementation challenges. Only data sources with sufficient completeness and consistency across these domains were included in the analysis.

### Analytical framework

The analysis was guided by a systems-thinking framework that conceptualizes digital health systems as dependent on interconnected infrastructure components. The framework focused on three interrelated domains: energy reliability, digital system performance, and health system outcomes. Energy reliability was considered in terms of availability, stability, and backup capacity, while digital system performance encompassed functionality of electronic medical records, data continuity, and system uptime. Health system outcomes were examined through indicators such as workflow efficiency, data quality, and service delivery capacity.

This framework draws on existing digital health implementation models that emphasize the importance of infrastructure readiness, interoperability, and system integration in achieving sustainable outcomes (1, 2, 8). In addition, insights from health information technology incident research were incorporated to understand how infrastructure failures contribute to system disruptions and potential risks to patient safety. Previous studies have demonstrated that failures in digital health systems often arise from complex interactions between software, hardware, and organizational environments, highlighting the critical role of foundational infrastructure in ensuring system reliability (14–16).

### Data analysis

Qualitative data from interviews and focus group discussions were analyzed using inductive thematic analysis, following established guidance (17, 18). Data were coded iteratively to identify patterns related to infrastructure constraints, system performance, and workflow disruptions. Codes were refined into higher-level themes aligned with the study objectives, including technical feasibility, economic viability, and health system readiness. The analytical process followed established qualitative research principles to ensure rigor, transparency, and consistency (17–19).

Quantitative data derived from observational checklists were analyzed descriptively, including indicators such as reliability of power supply, availability of backup systems, frequency of system downtime, and connectivity stability. These indicators provided contextual grounding for qualitative findings. A triangulation approach was used to integrate qualitative and quantitative data, enhancing the validity and robustness of the findings by comparing evidence across multiple data sources and methods (20). Rather than presenting detailed statistical analyses, the quantitative data were interpreted descriptively to reinforce the conceptual argument that digital health systems depend on reliable energy infrastructure.

The coding framework was developed iteratively through an inductive process. Initial codes were generated from a subset of transcripts and refined through repeated review to ensure consistency and relevance to the study objectives. Codes were then grouped into higher-order themes corresponding to technical feasibility, economic viability, and health system readiness. To enhance reproducibility, the coding structure and thematic categories were consistently applied across all data sources, and coding decisions were documented throughout the analysis process (18, 19).

To enhance analytical validity, findings were cross-checked across multiple data sources, including interviews, focus group discussions, and observational assessments, enabling methodological triangulation. Coding consistency was maintained through iterative review and discussion among the research team. Given the exploratory nature of the study, formal inter-coder reliability testing was not applied; however, efforts were made to ensure consistency and transparency in coding decisions through systematic documentation and review (21, 22).

### Conceptual synthesis

The integration of empirical findings and evidence from the literature was conducted through narrative synthesis, enabling the development of a coherent conceptual framework describing the “Data–Power Paradox.” Narrative synthesis is particularly suited to studies that aim to combine heterogeneous data sources and generate system-level insights in complex health environments (23). The synthesis process involved identifying patterns of misalignment between digital health investments and infrastructure readiness, mapping causal pathways linking energy constraints to system performance, and developing a conceptual model of solar-powered digital health architecture tailored to low-resource settings.

### Ethical considerations

The original Bright Health feasibility study received ethical approval from relevant institutional review boards in Kenya and Ethiopia (Approval No. SU-ISERC 3088/25). All participants provided informed consent prior to participation, and data were anonymized to ensure confidentiality and privacy.

The present study involved secondary analysis of de-identified data and did not involve direct interaction with participants. As such, no additional ethical approval was required. The study adhered to international ethical standards for research involving human participants, consistent with established guidelines (2, 8, 24).

## Conceptual framework

### Energy as a foundational health system input

Health systems are fundamentally dependent on a set of core infrastructural inputs that enable the delivery of safe, effective, and continuous care. Among these, energy is one of the most critical yet frequently overlooked components. Reliable electricity underpins the functioning of essential health services, including diagnostic equipment, cold chain systems, sterilization processes, lighting for clinical procedures, and increasingly, digital health infrastructure (2, 3, 8). In the absence of a stable energy supply, even well-resourced healthcare facilities experience disruptions in service delivery, reduced operational efficiency, and increased risks to patient safety.

In low- and middle-income countries, energy access remains highly uneven, particularly in rural and peri-urban areas, where health facilities often rely on unreliable grid connections, diesel generators, or intermittent alternative power sources (5). These limitations not only constrain the availability of essential services but also introduce systemic vulnerabilities that affect the broader functioning of health systems. From a systems perspective, energy can therefore be conceptualized as a foundational input that interacts with and supports all other components of healthcare delivery.

The increasing digitization of health systems further amplifies the importance of energy as a foundational enabler. Digital health technologies, including electronic medical records, telemedicine platforms, and health information systems, are inherently dependent on a continuous power supply for data storage, processing, and transmission. Without reliable energy, these systems are prone to interruptions that compromise data integrity, disrupt clinical workflows, and reduce user trust in digital health systems (1).

Evidence from health information technology research highlights how infrastructure instability can lead to cascading system failures. Studies examining incident reports in healthcare settings have shown that disruptions in hardware, software, and supporting infrastructure can result in significant operational challenges, including delays in care, loss of critical patient information, and compromised decision-making processes (16, 25–27). These findings underscore the interdependence between digital systems and their infrastructural environment, reinforcing the need to consider energy not as an external factor but as an integral component of digital health ecosystems.

From a conceptual standpoint, positioning energy as a foundational input to the health system enables a more holistic understanding of digital health implementation. It shifts the focus from isolated technological solutions to the broader infrastructural conditions required for their effective operation. This perspective is particularly important in low-resource settings, where addressing energy gaps may yield greater improvements in health system performance than introducing additional digital tools alone (1–3, 8).

To formalize the conceptual framework, this study defines three core constructs: (1) infrastructure reliability, comprising electricity availability, connectivity stability, and maintenance capacity; (2) digital health system performance, including system uptime, data continuity, and usability; and (3) health system outcomes, reflected in service continuity, workflow efficiency, and quality of care.

The framework specifies directional relationships between these constructs, whereby infrastructure reliability directly influences digital system performance, which in turn affects health system outcomes. In addition, infrastructure constraints exert indirect effects through feedback mechanisms, including workflow disruptions and reduced user trust in digital systems. This structure enables the conceptualization of digital health systems as infrastructure-dependent socio-technical systems rather than isolated technological interventions.

**Figure 1:**
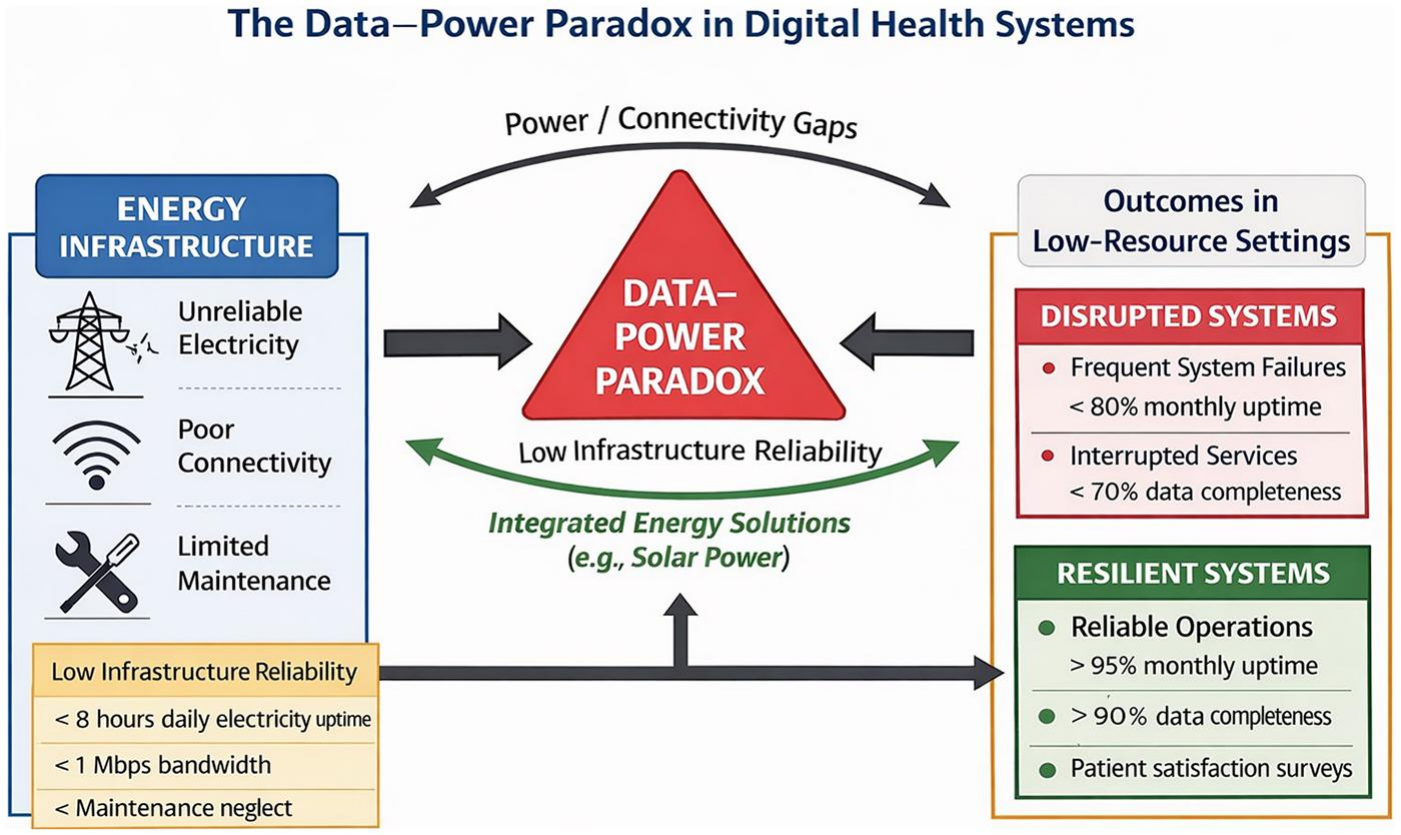
Conceptual model of the data–power paradox in digital health systems. The model illustrates the causal relationships between energy infrastructure conditions (e.g., electricity reliability, connectivity, maintenance capacity), digital health system performance (e.g., system uptime, data completeness), and health system outcomes (e.g., service continuity, quality of care). Infrastructure deficiencies lead to low system reliability, resulting in disrupted digital operations and fragmented care delivery. The model further incorporates indicative measurable thresholds (e.g., uptime percentage, data completeness rates) to demonstrate how system performance can be assessed. Integrated energy solutions, particularly solar-powered systems, act as an enabling intervention that improves infrastructure reliability, strengthens system performance, and supports resilient healthcare delivery.

To illustrate the interdependencies between energy infrastructure, digital health systems, and health system outcomes, a conceptual model of the Data–Power Paradox is presented in Figure 1. To enhance analytical clarity, the conceptual model specifies key variables and causal pathways linking infrastructure conditions to digital health system performance and health system outcomes. The model also incorporates indicative metrics to illustrate how these relationships may be operationalized and assessed in practice. These indicators can be operationalized in future empirical studies **using metrics such as system uptime logs, data completeness rates, outage frequency**, and continuity of service delivery across reporting periods.

### Digital health dependency on infrastructure

Digital health systems operate within complex socio-technical environments characterized by interdependencies between technology, infrastructure, and organizational processes. These systems rely not only on software and hardware components but also on stable supporting infrastructure, including electricity, connectivity, and maintenance capacity. The functionality and reliability of digital health systems are, therefore, contingent upon the stability of these underlying components (1, 2, 28).

In many low-resource settings, digital health initiatives have been implemented with limited infrastructure readiness. As a result, systems that are designed to improve efficiency and data management often fail to achieve their intended impact. Interruptions in power supply can lead to system shutdowns, data loss, and delays in accessing patient records, forcing healthcare providers to revert to manual processes. This cyclical pattern of adoption and abandonment undermines confidence in digital health technologies and limits their long-term sustainability (3).

The concept of infrastructure dependency is well established in information systems research, where system performance is understood to be shaped by interactions between technological components and their operating environment. In healthcare, this dependency is particularly pronounced due to the critical nature of clinical workflows and the need for timely and accurate information. Research on health information technology incidents has demonstrated that system failures often arise not from isolated technical faults but from misalignments between system design and infrastructural conditions (6, 29). These misalignments can manifest as configuration issues, software malfunctions, or workflow disruptions, all of which are exacerbated by unstable infrastructure.

The reliance of digital health systems on continuous connectivity further complicates this dependency. Many digital health systems are designed with the assumption of stable internet access, enabling real-time data synchronization and remote access to information. However, in settings where connectivity is intermittent or absent, these assumptions do not hold, resulting in reduced system functionality and user frustration. Emerging approaches, such as offline-first system design, seek to address these challenges by enabling local data storage and delayed synchronization, but their effectiveness remains contingent on reliable energy to power local servers and devices (30–32).

The dependency of digital health systems on infrastructure can therefore be understood as a critical determinant of their success or failure. In the absence of reliable energy and connectivity, digital health interventions risk becoming fragmented, underutilized, or entirely non-functional. This dependency lies at the core of the Data–Power Paradox, highlighting the need for integrated solutions that address both technological and infrastructural dimensions of health system strengthening (1, 3).

By conceptualizing digital health systems as infrastructure-dependent rather than standalone innovations, this study provides a foundation for rethinking implementation strategies in low-resource settings. It emphasizes the importance of aligning digital health investments with infrastructure capacity and sets the stage to explore how solar-powered energy solutions can mitigate these dependencies and enable more resilient, sustainable digital health ecosystems (2, 8, 13). This dependency can be conceptualized as a causal pathway in which instability in infrastructure inputs reduces system performance, which in turn impacts service delivery outcomes.

## Empirical insights from the bright health study

### Evidence of system failure due to power gaps

The empirical findings are presented below as structured observations, followed by interpretive insights that inform the conceptual framework. Empirical findings from the Bright Health feasibility study conducted across rural and peri-urban healthcare facilities in Kenya and Ethiopia reveal a consistent pattern of infrastructure-related disruptions affecting digital health systems. Across study sites, unreliable electricity supply emerged as a primary constraint limiting the effective use of digital health technologies. Frequent power outages, voltage fluctuations, and the absence of reliable backup systems resulted in intermittent functionality of electronic medical records and other digital health systems, often forcing healthcare providers to revert to paper-based processes.

These disruptions were not isolated technical issues but reflected systemic vulnerabilities embedded within the infrastructural environment. In several facilities, digital systems introduced to improve efficiency and data management were rendered partially or entirely nonfunctional during power interruptions. This resulted in fragmented patient records, duplication of work, and delays in clinical decision-making. Healthcare workers reported challenges in maintaining data continuity, particularly when transitions between digital and manual systems occurred during outages. Such inconsistencies compromised both workflow efficiency and the reliability of health information systems.

The findings illustrate how the effectiveness of digital health interventions is closely tied to the stability of the underlying infrastructure. Even in facilities where digital tools were available, and staff were trained in their use, the absence of a reliable energy supply undermined their practical utility. This aligns with broader evidence indicating that infrastructure constraints remain a major barrier to the successful implementation of digital health systems in low-resource settings (1, 3).

From a systems perspective, these observations reinforce the concept of infrastructure dependency discussed in the conceptual framework. Digital health systems do not fail solely due to deficiencies in software or user capacity but are often disrupted by external factors such as unstable power supply. This interdependence creates a cascade effect, where a failure in energy infrastructure propagates through digital systems and ultimately affects service delivery. Similar patterns have been documented in health information technology incident studies, which demonstrate how disruptions in supporting infrastructure can lead to broader system failures and operational inefficiencies (29, 33, 34).

In this context, the empirical findings provide concrete evidence of the Data–Power Paradox, in which investments in digital health technologies coexist with infrastructural conditions that prevent their effective use. The persistence of such conditions suggests that addressing energy reliability is not merely a technical enhancement but a prerequisite for realizing the benefits of digital health interventions.

### Role of solar systems in ensuring continuity

Alongside the challenges posed by unreliable grid electricity, the Bright Health study also highlighted the potential of solar-powered systems to mitigate infrastructure disruptions and support the continuous operation of digital health systems. Facilities that had access to solar energy solutions, either as primary or supplementary power sources, demonstrated improved system uptime and greater consistency in the use of digital health tools.

Solar-powered systems provided a decentralized and relatively stable source of electricity, enabling health facilities to maintain essential operations during grid outages. This included not only powering digital devices and servers but also supporting broader clinical functions such as lighting, refrigeration, and communication systems. As a result, healthcare providers in these settings were better able to sustain digital workflows, maintain data continuity, and deliver uninterrupted services.

The introduction of solar energy also influenced user confidence in digital systems. In environments characterized by frequent power interruptions, healthcare workers often developed workarounds that prioritized manual processes due to uncertainty regarding system availability. In contrast, the presence of reliable solar power reduced reliance on such workarounds and encouraged more consistent use of digital health systems. This shift highlights the role of infrastructure stability in shaping user behavior and technology adoption.

Importantly, the benefits of solar-powered systems extended beyond immediate operational continuity. By providing a more predictable energy supply, these systems created an enabling environment for integrating additional digital health functionalities, including offline data storage and delayed-synchronization mechanisms. This supports the broader argument that energy infrastructure is not only a supporting component but a critical enabler of digital health system resilience.

These findings are consistent with existing evidence demonstrating that solar electrification can enhance healthcare service delivery in low-resource settings by improving reliability, reducing operational disruptions, and enabling the use of digital technologies (13, 35, 36). However, the empirical insights from the Bright Health study further contribute to this body of knowledge by illustrating how energy solutions directly affect digital system performance and user practices in real-world healthcare environments.

While these findings may appear intuitive, the analysis reveals a consistent and systematic relationship between infrastructure instability and digital health system performance across diverse facility contexts. Rather than isolated operational challenges, power and connectivity gaps function as structural constraints that shape system reliability, data continuity, and workflow integration.

#### Conceptual interpretation

This suggests that infrastructure limitations are not peripheral but central determinants of digital health system functionality. Importantly, the findings extend beyond descriptive observation by demonstrating how these constraints produce cascading effects across multiple levels of the health system, including clinical workflows, data management, and service delivery. This reinforces the argument that digital health interventions in low-resource settings cannot be evaluated independently of the infrastructural environments in which they operate.

A summary of key empirical observations and their corresponding conceptual interpretations is presented in Table 1.

**Table 1:** Summary of empirical observations and corresponding interpretations.

Empirical Observation	Conceptual Interpretation
Frequent power outages disrupt system access	Infrastructure instability reduces system reliability
Facilities revert to paper-based processes	Digital systems lack resilience under infrastructure constraints
Stable energy environments show consistent system use	Energy reliability enables sustained digital workflows

## System model: solar-powered digital health architecture

### Offline-first digital health systems

The effective implementation of digital health systems in low-resource settings requires a departure from conventional models that assume continuous connectivity and stable infrastructure. Instead, there is a need for system architectures explicitly designed to operate under intermittent power supply and limited network availability (37). An offline-first approach is a critical design principle in this context, enabling digital health systems to operate without continuous internet connectivity while maintaining data integrity and usability (1, 15, 38, 39).

Offline-first systems prioritize local data storage and processing, allowing healthcare providers to capture, access, and update patient information in real time without reliance on external servers. Data generated at the point of care are stored on local devices or facility-based servers and are synchronized with central or cloud-based systems when connectivity becomes available. This approach reduces the risk of data loss during power or network interruptions and ensures continuity of care even in constrained environments (1, 13, 40, 41).

#### Empirical observation

The empirical findings from the Bright Health study underscore the importance of such an approach. Facilities experiencing frequent power outages and unstable connectivity reported disruptions in accessing digital systems, often necessitating a fallback to paper-based processes. In contrast, environments supported by more stable energy sources demonstrated improved system reliability and greater consistency in digital workflows. These observations highlight that offline-first functionality alone is insufficient without reliable energy infrastructure, as local servers and devices still require continuous power to operate effectively (3, 8).

By integrating offline-first capabilities with stable energy sources, particularly solar-powered systems, digital health systems can achieve greater resilience. This integration enables healthcare facilities to maintain uninterrupted access to patient data, support clinical decision-making, and reduce reliance on manual processes (36). The offline-first model, therefore, serves as a critical bridge between technological design and infrastructural realities, aligning system functionality with the constraints of low-resource settings.

### Hybrid connectivity: SIM and satellite integration

While offline-first systems address the challenges of intermittent connectivity, the ability to synchronize data with centralized platforms remains essential to broader health system functions, including reporting, surveillance, and care coordination (1, 2, 42). In low-resource settings, where network infrastructure is often unreliable or unevenly distributed, hybrid connectivity models offer a pragmatic solution to ensure data exchange and system integration (43).

Hybrid connectivity uses multiple communication channels, such as mobile networks (e.g., 3G/4G SIM-based connectivity) and satellite links, to enable data transmission between local systems and cloud-based platforms. Mobile networks provide a cost-effective and widely available means of connectivity in many regions, although their performance may vary depending on geographic and infrastructural factors (44). Satellite connectivity, while more resource-intensive, offers an additional layer of reliability in remote or underserved areas where terrestrial networks are limited or unavailable (43).

**Figure 2:**
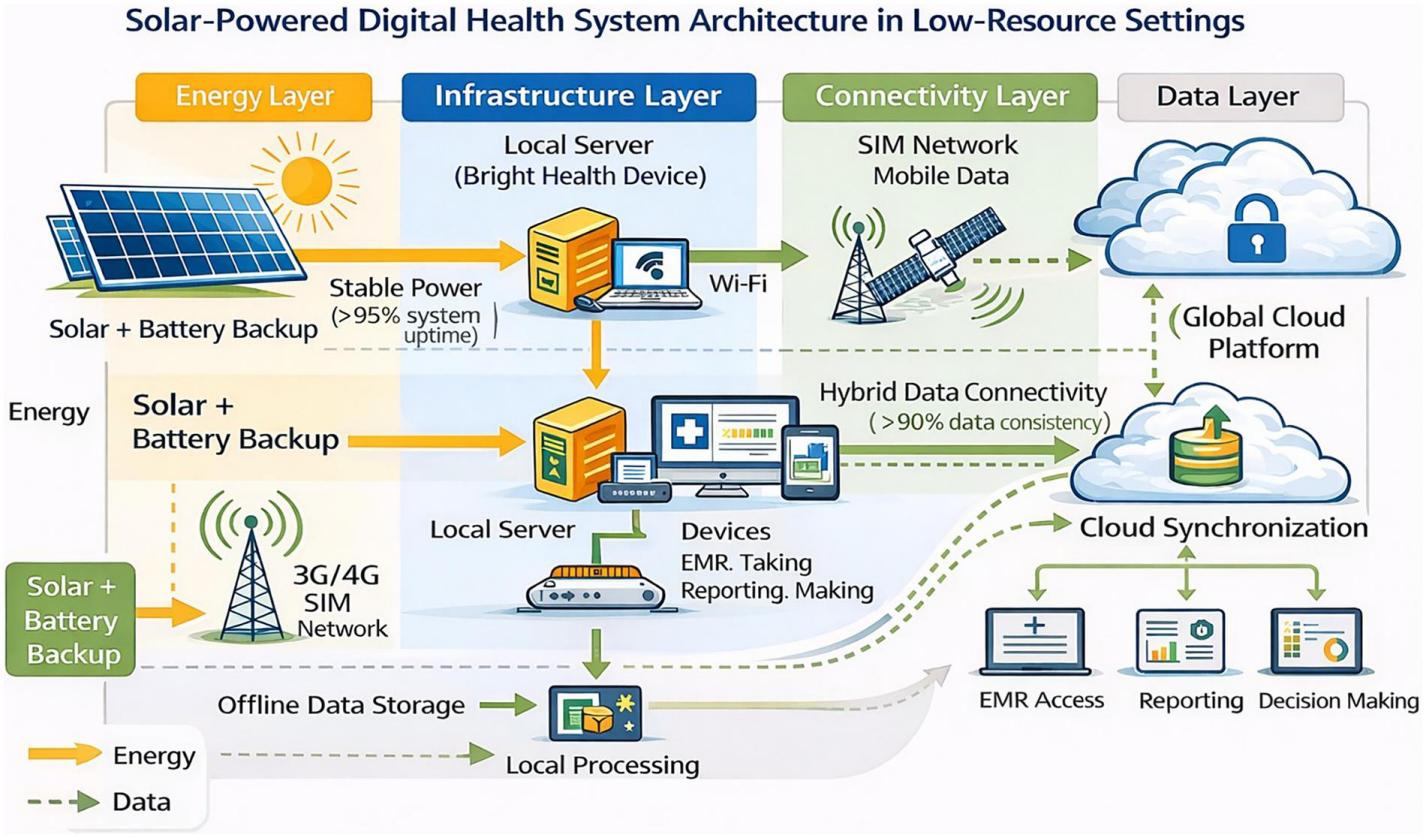
System architecture of a solar-powered digital health system in low-resource settings. The figure presents a multi-layered architecture showing how solar energy, supported by battery storage, powers local digital health infrastructure, including facility-based servers and user devices. It illustrates the interaction between offline-first data processing, in which patient information is stored and accessed locally, and hybrid connectivity mechanisms, such as mobile networks and satellite links, that enable periodic synchronization with cloud-based systems. The model highlights how integrated energy and connectivity solutions support continuous access to electronic medical records, reporting functions, and clinical decision-making despite infrastructural constraints.

The system architecture was refined to explicitly reflect directional energy and data flows, reduce visual redundancy, and align more closely with empirically observed system dependencies. The system architecture illustrated in Figure 2 demonstrates how these connectivity layers can be integrated with local digital infrastructure and energy systems to support continuous data flow. Data generated and stored locally via offline-first systems can be periodically synchronized with cloud platforms when connectivity is available, ensuring that information is up to date and accessible at higher levels of the health system. This approach balances the need for real-time data access at the facility level with the broader requirements of health system integration and reporting (1).

Importantly, the effectiveness of hybrid connectivity models remains closely linked to the availability of reliable energy. Both mobile and satellite communication systems depend on powered devices, network equipment, and local servers to function. As such, integrating solar energy systems provides a critical enabling layer that supports not only local system operation but also connectivity and data exchange (8).

By combining solar-powered energy infrastructure, offline-first system design, and hybrid connectivity mechanisms, the proposed architecture addresses the key limitations identified in the empirical findings and conceptual framework. It provides a scalable and adaptable model for implementing digital health systems that are resilient to infrastructural constraints, thereby directly addressing the challenges posed by the Data–Power Paradox.

While the proposed architecture is conceptually coherent, its full implementation may be constrained by context-specific factors, including financial resources, technical capacity, and infrastructure availability. In many low-resource settings, deployment is likely to occur in a phased or modular manner, with components such as solar energy systems, offline-first functionality, and connectivity solutions implemented incrementally. Key challenges include upfront investment costs, maintenance of energy systems, and availability of trained technical personnel. As such, the architecture should be interpreted as a flexible reference model that can be adapted to local conditions rather than as a uniform implementation blueprint. These constraints may vary significantly across settings, but phased implementation strategies and alignment with local resource capacities can improve feasibility and sustainability.

The proposed architecture is derived from empirical observations and partially implemented components observed in the Bright Health feasibility study; however, it has not yet been deployed or evaluated as a fully integrated system (Kenya and Ethiopia). It, therefore, represents a conceptual synthesis informed by real-world implementation insights rather than a fully validated system model.

## Discussion

The findings of this study extend beyond descriptive observations, demonstrating that infrastructure constraints serve as system-level determinants rather than peripheral challenges. While prior research has acknowledged the role of electricity and connectivity in shaping digital health implementation (4), these factors have often been treated as contextual barriers. In contrast, this study shows that infrastructure reliability directly structures system performance, influencing uptime, data continuity, and workflow integration. This perspective aligns with socio-technical systems theory, which emphasizes the interdependence between technological systems and their operational environments (28), and provides a conceptual basis for the Data–Power Paradox. While the empirical analysis provides structured observational insights, it is primarily descriptive and intended to support conceptual interpretation rather than to establish causal inference.

This study can also be situated within the broader implementation science literature, which emphasizes the importance of contextual factors in determining the success of health interventions. Established frameworks such as the Consolidated Framework for Implementation Research (CFIR) and RE-AIM highlight domains including inner setting, infrastructure, and implementation context as critical determinants of intervention outcomes (45–49). However, these frameworks often treat infrastructure as one of several contextual variables. In contrast, the present study extends this perspective by positioning energy infrastructure as a foundational, system-defining component that directly shapes the performance of digital health systems. In doing so, the Data–Power Paradox complements existing frameworks by providing a more explicit account of infrastructural dependencies and by challenging the implicit assumption of stable operational environments in digital health implementation.

Beyond implementation science, the proposed framework can be further understood through broader theoretical perspectives. From a socio-technical systems perspective, digital health systems are shaped by the interaction between technical components and their infrastructural and organizational environments (14, 28). Infrastructure theory further emphasizes that foundational systems, such as energy, are often invisible yet critical enablers that determine the functionality of higher-level services (50). In addition, digital ecosystem models highlight the interdependence between technological platforms, connectivity layers, and supporting infrastructure in enabling system-wide functionality and scalability (51).

By integrating these perspectives, the Data–Power Paradox provides a more comprehensive theoretical account of how infrastructural conditions shape digital health system performance. It extends existing approaches by explicitly foregrounding energy as a core infrastructural dependency and situating digital health systems within a broader ecosystem of interdependent components (1, 52, 53).

Unlike existing socio-technical and implementation science frameworks, which typically treat infrastructure as a component within broader contextual domains, this study explicitly positions energy as a foundational, system-defining input that directly shapes the functionality of digital health systems (5, 38, 44). The novelty of the Data–Power Paradox lies in its articulation of infrastructure not as a background condition but as a primary determinant of system performance, supported by empirical observations from real-world implementation contexts. In doing so, the framework moves beyond extending existing models by introducing a more explicit infrastructure-centered lens for understanding digital health system success and failure in low-resource settings.

The proposed architecture builds on established components, including offline-first system design, hybrid connectivity models, and solar-powered infrastructure, which have been explored individually in prior studies. The novelty of this work lies in integrating these components into a unified, infrastructure-centered system architecture that explicitly addresses their interdependencies in low-resource health system contexts.

The proposed framework also contributes to innovation in current user-centered design (UCD) approaches for digital health implementation. Existing UCD models have primarily focused on usability, workflow integration, and alignment with user needs at the interface and service levels. Our findings suggest that in low-resource settings, these design priorities are insufficient unless they also account for infrastructural dependencies that directly shape user experience and system usability. By incorporating energy reliability, connectivity constraints, and service continuity as core design parameters, the Data–Power Paradox extends current UCD approaches toward a more infrastructure-aware model of digital health design, better suited to resource-constrained implementation environments.

More broadly, the Data–Power Paradox can be understood as reflecting structural interdependencies across multiple system layers, including energy, connectivity, and data infrastructures. These layers are tightly coupled, such that disruptions in one domain propagate across the system, affecting overall functionality and performance. This perspective aligns with emerging approaches in complex adaptive systems and infrastructure interdependency frameworks, which emphasize non-linear dynamics, system bottlenecks, and cross-layer dependencies in real-world implementation environments (1, 43, 54, 55). By framing digital health systems within these interdependent layers, this study extends beyond a specific architectural proposal and offers a generalizable systems-level insight into implementation challenges in low-resource settings.

### Transformational role of energy integration in health systems

This study highlights the critical yet under-recognized role of energy infrastructure in enabling the effective implementation of digital health systems in low-resource settings. By conceptualizing energy as a foundational component of health system functionality, the findings challenge prevailing approaches that prioritize technological innovation without adequately addressing infrastructural readiness. The empirical insights from the Bright Health study demonstrate that digital health systems are highly sensitive to disruptions in energy supply, with power instability leading to system failures, workflow inefficiencies, and reduced data reliability. While the empirical observations are consistent with existing evidence on infrastructure challenges in low-resource settings, this study advances the literature by conceptualizing these challenges as an interconnected system-level phenomenon rather than isolated barriers. The Data–Power Paradox provides a unifying explanation for why digital health systems often fail to deliver expected benefits despite the availability of technology, highlighting the structural dependence of digital systems on energy and connectivity infrastructure.

These findings align with broader evidence indicating that infrastructure constraints remain a significant barrier to the successful scaling of digital health interventions in low- and middle-income countries (1, 3). While considerable investments have been made in developing and deploying digital health technologies, less attention has been given to the underlying systems required to sustain their operation. This imbalance contributes to the persistence of the Data–Power Paradox, where digital tools are introduced into environments that are not adequately equipped to support them.

This study contributes by reframing infrastructure not as a background condition but as a core component of digital health system design and evaluation. Previous studies have documented challenges related to electricity and connectivity (2–4, 8), but have largely treated them as contextual barriers. In contrast, this study demonstrates that these factors actively shape system behavior, influencing uptime, data reliability, and user engagement. This perspective aligns with socio-technical systems theory, which emphasizes the interdependence of technological and infrastructural components (27, 28, 56).

The integration of solar-powered energy systems offers a transformative pathway to address this challenge. By providing a decentralized and reliable source of electricity, solar solutions can stabilize the operational environment of healthcare facilities, enabling consistent use of digital health systems. The findings from this study suggest that such integration not only improves system uptime but also enhances user confidence and promotes sustained adoption of digital technologies (13, 57–59). This is particularly important in settings where repeated system failures have led to reliance on manual workarounds and reduced trust in digital solutions.

From a systems perspective, the integration of energy and digital health infrastructure represents a shift towards more holistic and resilient models of healthcare delivery. Rather than treating energy access as an external constraint, this approach embeds it within the design and implementation of digital health systems. Similar arguments have been made in the broader health systems literature, which emphasizes the need for integrated approaches that address multiple system components simultaneously to achieve sustainable improvements in health outcomes (2, 8, 60, 61).

This finding extends existing literature by demonstrating that energy infrastructure is not merely an enabling factor but a core determinant of digital health system performance. While previous studies have emphasized the importance of digital health technologies, fewer have explicitly linked system reliability to energy availability in a structured manner (1, 55).

### Implications for sustainable development goals and health equity

The findings of this study have important implications for global health policy, particularly in relation to the Sustainable Development Goals (SDGs). The integration of energy and digital health systems directly contributes to SDG 3 (Good Health and Well-being) and SDG 7 (Affordable and Clean Energy) by improving access to quality healthcare services while promoting sustainable energy solutions (8, 62, 63). By addressing the infrastructural barriers that limit the effectiveness of digital health interventions, integrated approaches can enhance the equity and inclusiveness of health systems.

In many low-resource settings, disparities in access to infrastructure contribute to inequities in healthcare delivery, with rural and remote populations disproportionately affected by unreliable energy and limited connectivity (5, 43, 64). Digital health technologies have often been proposed as a means to bridge these gaps; however, without addressing underlying infrastructural constraints, such technologies risk reinforcing existing inequalities (1, 65). The findings of this study suggest that integrating solar-powered energy solutions with digital health systems can help mitigate these disparities by enabling more consistent and reliable service delivery across diverse settings.

Furthermore, the proposed system architecture provides a scalable model for strengthening health systems in resource-constrained environments. By combining offline-first system design with hybrid connectivity and renewable energy sources, the model addresses key challenges related to infrastructure dependency and system resilience. This approach aligns with global strategies that emphasize the importance of context-appropriate and sustainable solutions in advancing digital health and achieving universal health coverage (3).

More broadly, this study contributes to ongoing discussions on the need for cross-sectoral collaboration in health system strengthening. The intersection of energy and health highlights the importance of coordinated efforts between the health sector, energy providers, technology developers, and policymakers. Addressing the Data–Power Paradox requires integrated planning and investment strategies that recognize the interdependence of these sectors.

These findings support broader calls for integrated approaches to health system strengthening that align digital health strategies with infrastructure development (3). Without such alignment, digital health interventions risk reinforcing existing inequities rather than addressing them.

### Limitations of the study

Despite its contributions, this study has limitations that should be considered. The empirical insights are derived from a limited number of facilities in Kenya and Ethiopia, which may not fully represent the diversity of contexts in other low-resource settings. Additionally, the study focuses primarily on infrastructure-related factors and does not extensively examine other determinants of digital health adoption, such as policy environments, financing mechanisms, and user-level factors. Future research should explore these dimensions and further validate the proposed system model across different settings.

The generalizability of the findings should therefore be considered in relation to contextual factors, including variations in energy infrastructure, health system organization, and levels of digital health maturity across different LMIC regions. While the study identifies system-level relationships that are likely applicable across similar resource-constrained settings, the magnitude and manifestation of these effects may vary depending on local infrastructural conditions and implementation environments. The findings are most transferable to settings characterized by unreliable electricity supply, limited connectivity, and emerging digital health adoption. Future research should validate and adapt the proposed framework across diverse geographical and health system contexts to strengthen its external applicability.

The study is based on a single feasibility study, which may introduce contextual bias and limit generalizability. In addition, the analysis is primarily descriptive and does not include longitudinal or comparative data, and the proposed conceptual framework has not yet been empirically validated across multiple settings.

## Conclusion

This study introduces the concept of the Data–Power Paradox to highlight a critical gap in the implementation of digital health systems in low-resource settings. While digital technologies have been widely promoted as tools for strengthening health systems, their effectiveness remains fundamentally constrained by the availability and reliability of underlying infrastructure, particularly energy. By integrating empirical insights from the Bright Health study with a systems-level conceptual framework, this paper demonstrates that energy is not a peripheral consideration but a foundational enabler of digital health functionality.

The findings show that unreliable power supply undermines the performance, continuity, and adoption of digital health systems, leading to fragmented workflows and reduced data reliability. In contrast, integrating solar-powered energy solutions can significantly enhance system stability, support continuous digital operations, and improve overall service delivery. These insights underscore the importance of aligning digital health investments with infrastructural capacity to ensure sustainable and effective implementation.

The proposed solar-powered digital health architecture, combining offline-first system design with hybrid connectivity mechanisms, offers a practical, scalable approach to addressing infrastructure constraints. By enabling resilient system performance in environments with limited connectivity and unstable power supplies, this model offers a pathway to overcoming the challenges associated with the Data–Power Paradox.

Ultimately, this study calls for a shift in how digital health interventions are conceptualized and implemented. Rather than treating digital technologies as standalone solutions, there is a need to adopt integrated approaches that recognize the interdependence of energy, infrastructure, and health systems. Such approaches are essential for advancing equitable access to healthcare, supporting sustainable development goals, and ensuring that digital health innovations deliver meaningful and lasting impact in low-resource settings.

## Data Availability

The original contributions presented in the study are included in the article/Supplementary Material, further inquiries can be directed to the corresponding author.
